# Reply to Bruschi, F.; Casini, G. New Models for the Study of Ocular Toxoplasmosis. Comment on “Ashander et al. Human Retinal Organoid Model of Ocular Toxoplasmosis. *Pathogens* 2025, *14*, 286”

**DOI:** 10.3390/pathogens15040358

**Published:** 2026-03-27

**Authors:** Liam M. Ashander, Justine R. Smith

**Affiliations:** Flinders Health and Medical Research Institute, College of Medicine and Public Health, Flinders University, Bedford Park, Adelaide, SA 5042, Australia; liam.ashander@flinders.edu.au

We welcome the comment [1] written by Dr. Bruschi and Dr. Casini regarding our article, “Human retinal organoid model of ocular toxoplasmosis” [2]. This article represented the first report of ocular toxoplasmosis modelled using human retinal organoids. As explained in the Introduction of our article, we sought to demonstrate that infection of retinal organoids with the Apicomplexan parasite, *Toxoplasma gondii*, was feasible, that this infection progressed over time, and that cells within the organoids responded to the infection. Dr. Bruschi and Dr. Casini have rightly pointed out that it is not possible to ascertain which retinal layers or cell populations are infected with *T. gondii* in our studies. As noted in the Discussion of our article, we anticipate that future studies by ourselves and other research teams will address this important question.

Dr. Bruschi and Dr. Casini discuss the value of experimental ocular toxoplasmosis in mice. In the Discussion of our article, we described several seminal studies on the roles of chemokines in ocular toxoplasmosis that used this model. Nevertheless, as Mestas and Hughes [3] summarize in their highly cited review, “Of mice and not men: differences between mouse and human immunology”, there are numerous differences in mouse and human innate and adaptive immune systems. The differences between the human and mouse host response to *T. gondii* infection also cannot be overlooked. In the context of cell autonomous responses to the parasite, the mouse is singly reliant on immunity-related GTPases for parasite control, whereas this system has largely been lost in humans [4,5]. A recent report suggests that nitric oxide production, a major mechanism of parasite growth restriction in mice, may have the opposite function in humans [6]. More broadly, rodents are a key intermediate host in the parasite life cycle; humans are not. In comparing the innate immune responses of mice and humans to *T. gondii*, Sher et al. [7] indeed state, “rodents are more than just a convenient experimental model for studying infection with this protozoan pathogen.” It is clear from the context, however, that this is not an endorsement of experimental rodent models in research on toxoplasmosis, but an admonition that rodent research must be interpreted with caution, as millennia of co-evolution have shaped the parasite and the response of the mouse in ways distinct from that of the human.

All experimental systems have strengths and limitations. Accompanied by relevant references, we discussed the advantages and disadvantages of retinal two-dimensional cultured cell and tissue explant models in the Introduction of our article. In addition to their obvious strengths, retinal organoid models have limitations too: culture is time- and cost-intensive, and the resulting organoids are often heterogeneous, although efforts to tackle these issues are progressing [8]. One benefit of ex vivo models, such as the mouse retinal explant model reported by Dr. Bruschi’s and Dr. Casini’s teams, with correction [9], is the presence of microglia. First-generation retinal organoids generated from induced pluripotent stem cells lack integrated microglia and so cannot be used to study their role in toxoplasmic retinal infections, although new methodology is addressing this limitation [10]. Given the high prevalence of ocular toxoplasmosis and the substantial visual morbidity and lack of curative drug treatments [11,12], new models that facilitate discovery research around this disease continue to be welcomed.

## References

[1] Bruschi F., Casini G. (2026). New Models for the Study of Ocular Toxoplasmosis. Comment on Ashander et al. Human Retinal Organoid Model of Ocular Toxoplasmosis. *Pathogens* 2025, *14*, 286. Pathogens.

[2] Ashander L.M., Lidgerwood G.E., Lumsden A.L., Furtado J.M., Pebay A., Smith J.R. (2025). Human Retinal Organoid Model of Ocular Toxoplasmosis. Pathogens.

[3] Mestas J., Hughes C.C. (2004). Of mice and not men: Differences between mouse and human immunology. J. Immunol..

[4] Könen-Waisman S., Howard J.C. (2007). Cell-autonomous immunity to *Toxoplasma gondii* in mouse and man. Microbes Infect..

[5] Bekpen C., Hunn J.P., Rohde C., Parvanova I., Guethlein L., Dunn D.M., Glowalla E., Leptin M., Howard J.C. (2005). The interferon-inducible p47 (IRG) GTPases in vertebrates: Loss of the cell autonomous resistance mechanism in the human lineage. Genome Biol..

[6] Bando H., Lee Y., Sakaguchi N., Pradipta A., Ma J.S., Tanaka S., Cai Y., Liu J., Shen J., Nishikawa Y. (2018). Inducible Nitric Oxide Synthase Is a Key Host Factor for Toxoplasma GRA15-Dependent Disruption of the Gamma Interferon-Induced Antiparasitic Human Response. mBio.

[7] Sher A., Tosh K., Jankovic D. (2017). Innate recognition of *Toxoplasma gondii* in humans involves a mechanism distinct from that utilized by rodents. Cell. Mol. Immunol..

[8] Harkin J., Peña K.H., Gomes C., Hernandez M., Lavekar S.S., So K., Lentsch K., Feder E.M., Morrow S., Huang K.-C. (2024). A highly reproducible and efficient method for retinal organoid differentiation from human pluripotent stem cells. Proc. Natl. Acad. Sci. USA.

[9] Rodriguez Fernandez V., Amato R., Piaggi S., Pinto B., Casini G., Bruschi F. (2024). A New Ex Vivo Model Based on Mouse Retinal Explants for the Study of Ocular Toxoplasmosis. Pathogens.

[10] Usui-Ouchi A., Giles S., Harkins-Perry S., Mills E.A., Bonelli R., Wei G., Ouchi Y., Ebihara N., Nakao S., Friedlander M. (2023). Integrating human iPSC-derived macrophage progenitors into retinal organoids to generate a mature retinal microglial niche. Glia.

[11] Ferreira L.B., Furtado J.M., Charng J., Franchina M., Matthews J.M., Molan A.A.L., Hunter M., Mackey D.A., Smith J.R. (2022). Prevalence of toxoplasmic retinochoroiditis in an Australian adult population: A community-based study. Ophthalmol. Retin..

[12] Smith J.R., Ashander L.M., Arruda S.L., Cordeiro C.A., Lie S., Rochet E., Belfort R., Furtado J.M. (2021). Pathogenesis of ocular toxoplasmosis. Prog. Retin. Eye Res..

